# Case Report: Pansynostosis, Chiari I Malformation and Syringomyelia in a Child With Frontometaphyseal Dysplasia 1

**DOI:** 10.3389/fped.2021.574402

**Published:** 2021-07-01

**Authors:** Jaewon Kim, Dong-Woo Lee, Dae-Hyun Jang

**Affiliations:** Department of Rehabilitation Medicine, College of Medicine, The Catholic University of Korea, Seoul, South Korea

**Keywords:** frontometaphyseal dysplasia 1, otopalatodigital spectrum disorder, syringomyelia, Chiari I malformation, FLNA gene mutation, pansynostosis

## Abstract

Frontometaphyseal dysplasia 1 (FMD1) is a rare otopalatodigital spectrum disorder (OPDSD) that is inherited as an X-linked trait and it is caused by gain-of-function mutations in the *FLNA*. It is characterized by generalized skeletal dysplasia, and craniofacial abnormalities including facial dysmorphism (supraorbital hyperostosis, hypertelorism, and down-slanting palpebral fissures). The involvement of the central nervous system in patients with OPDSD is rare. Herein, we present the case of a 12-year-old boy with facial dysmorphism, multiple joint contractures, sensorineural hearing loss, scoliosis, craniosynostosis, and irregular sclerosis with hyperostosis of the skull. Brain and whole-spine magnetic resonance imaging revealed Chiari I malformation with extensive hydrosyringomyelia from the C1 to T12 levels. Targeted next-generation sequencing identified a hemizygous pathologic variant (c.3557C>T/p.Ser1186Leu) in the *FLNA*, confirming the diagnosis of FMD1. This is the first report of a rare case of OPDSD with pansynostosis and Chiari I malformation accompanied by extensive syringomyelia.

## Introduction

Otopalatodigital spectrum disorder (OPDSD), which is characterized by skeletal dysplasia, includes six disorders, OPD type 1 (OMIM #311300) and 2 (OMIM #304120), frontometaphyseal dysplasia 1 (FMD1; OMIM #305620), Melnick–Needles syndrome (MNS; OMIM #309350), digitocutaneous dysplasia (DCD; OMIM #300244), and a disorder characterized by keloid scarring, joint contractures, and cardiac valvulopathy (1, 2). Patients with these diseases commonly have mutations in the filamin A (*FLNA*) (3). The *FLNA* is located on chromosome Xq28 and is known to cause OPDSD (4). It encodes a 280-kDa cytoskeletal protein containing an actin-binding domain, which is important for cytoplasmic signaling. This protein interacts with transmembrane receptors or intracellular signaling molecules as well as filamentous actin and anchors transmembrane proteins to the actin cytoskeleton (5–8). The diseases accompanying the pathogenic variant of *FLNA* are termed X-linked filaminopathies. Disorders with a pathogenic variant of *FLNA* are classified as either a gain-of-function or a loss-of-function *FLNA* disorder, and OPDSD is classified as a gain-of-function disorder (2). Patients with OPDSD show various clinical manifestations, such as skeletal dysplasia, craniofacial dysmorphism, or sensorineural and/or conductive hearing loss (3, 9). Although clinical manifestations vary in females, the severity of this disease is generally milder in females than in males (10). The cardinal criteria for the diagnosis of FMD1 are craniofacial abnormalities including facial dysmorphism such as supraorbital hyperostosis, hypertelorism, and down-slanting palpebral fissures (2, 11). FMD2 caused by *MAP3K7* mutations and FMD3 caused by *TAB2* mutations exhibit clinical features similar to those of FMD1, but show autosomal dominant trait (2, 12, 13). Among the clinical manifestations of OPDSD, the involvement of central nervous system (CNS) is relatively rare. The presence of Chiari I malformation (CM1), cerebellar hypoplasia, hydrocephalus, and cerebellar tonsillar herniation have been previously reported in a few patients with OPDSD (14–17); however, these cases were only clinically reported and did not undergo genetic confirmation. Herein, we report for the first case of FMD1, with pansynostosis and Chiari I malformation accompanied by extensive syringomyelia.

## Case Presentation

### Clinical Presentation

**Figure 1:**
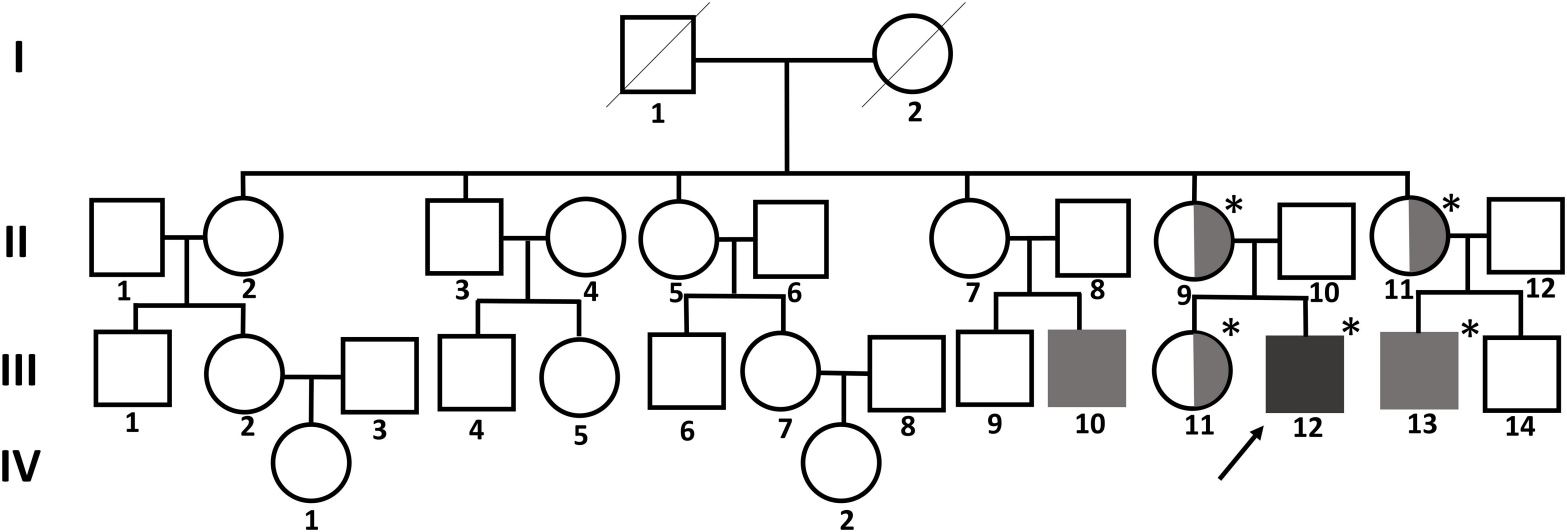
Pedigree chart of the family with FMD1. The asterisk indicates people tested for the *FLNA* pathogenic variant. The affected members are indicated by gray shading. Symbols divided into halves indicate heterozygous carriers of FMD1. The arrow indicates the proband.

A 12-year-old boy visited our clinic with complaints of back pain since 2 years. His medical history included bilateral sensorineural hearing loss and multiple joint contractures on both wrists and fingers, most prominently in the proximal interphalangeal joint of the left fifth finger. He has two male cousins who resemble him in appearance. His parents and sister show no obvious anomalies (Figure 1).

Physical examination revealed facial dysmorphism with micrognathia, frontal bossing, prominent supraorbital ridge, and hypertelorism. Furthermore, hypodontia, hypoplasia of the left fifth digital phalanx and great toes, and thoracic scoliosis were noted. The intrinsic muscles of both hands were graded as 2/5 based on the manual muscle test. There was no sensory impairment. Knee and ankle jerks were hyperreflexic and ankle clonus was evoked. His height was 154 cm (63.9 percentile).

**Figure 2:**
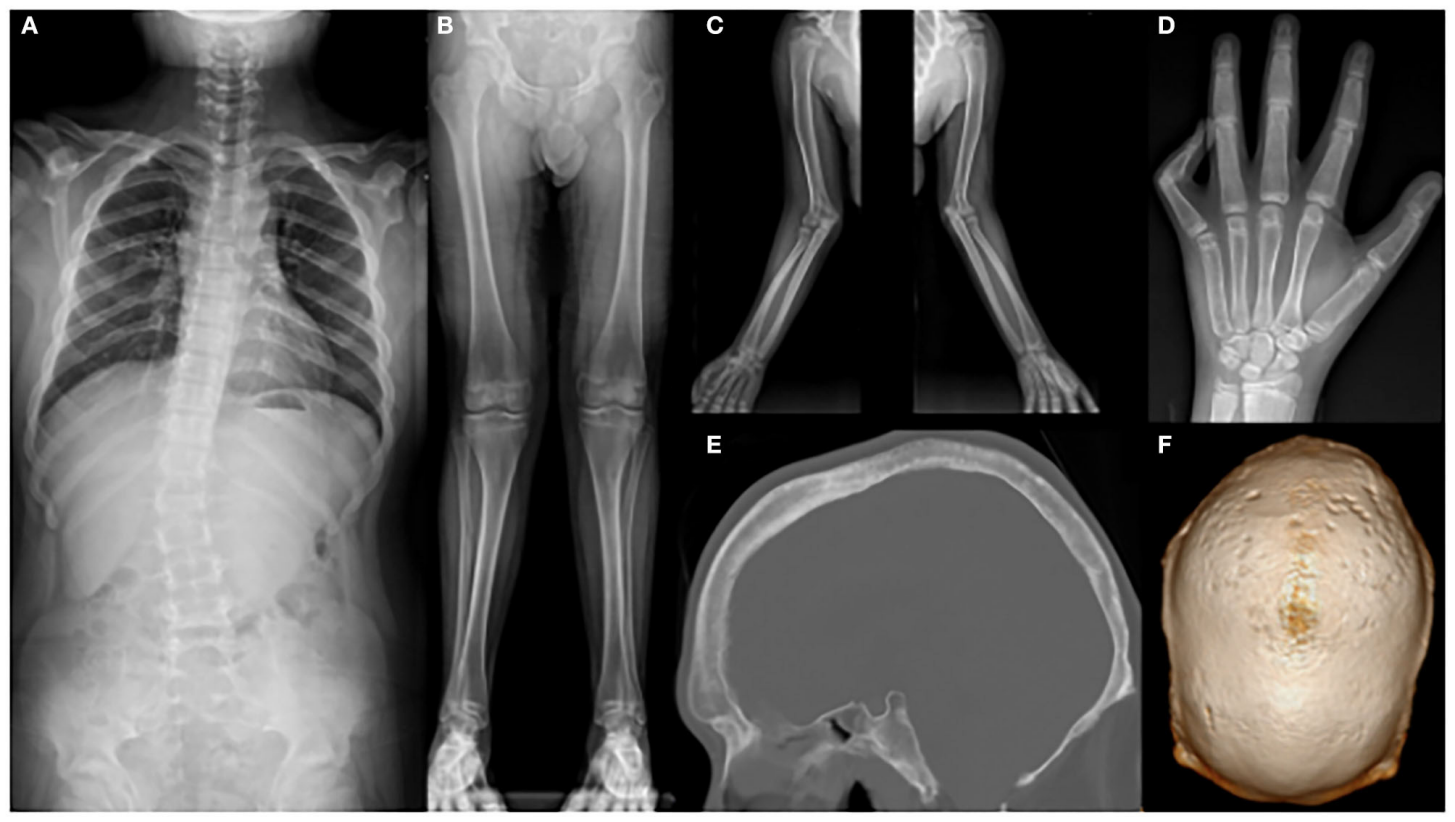
X-ray showing **(A)** scoliosis of the whole spine; **(B)** mild bowing of the tibia and **(C)** humerus; **(D)** flexion contracture of the left fifth proximal phalangeal joint; **(E)** hyperostosis, irregular sclerosis, frontal bone thickening, and protrusion; and **(F)** craniosynostosis.

**Figure 3:**
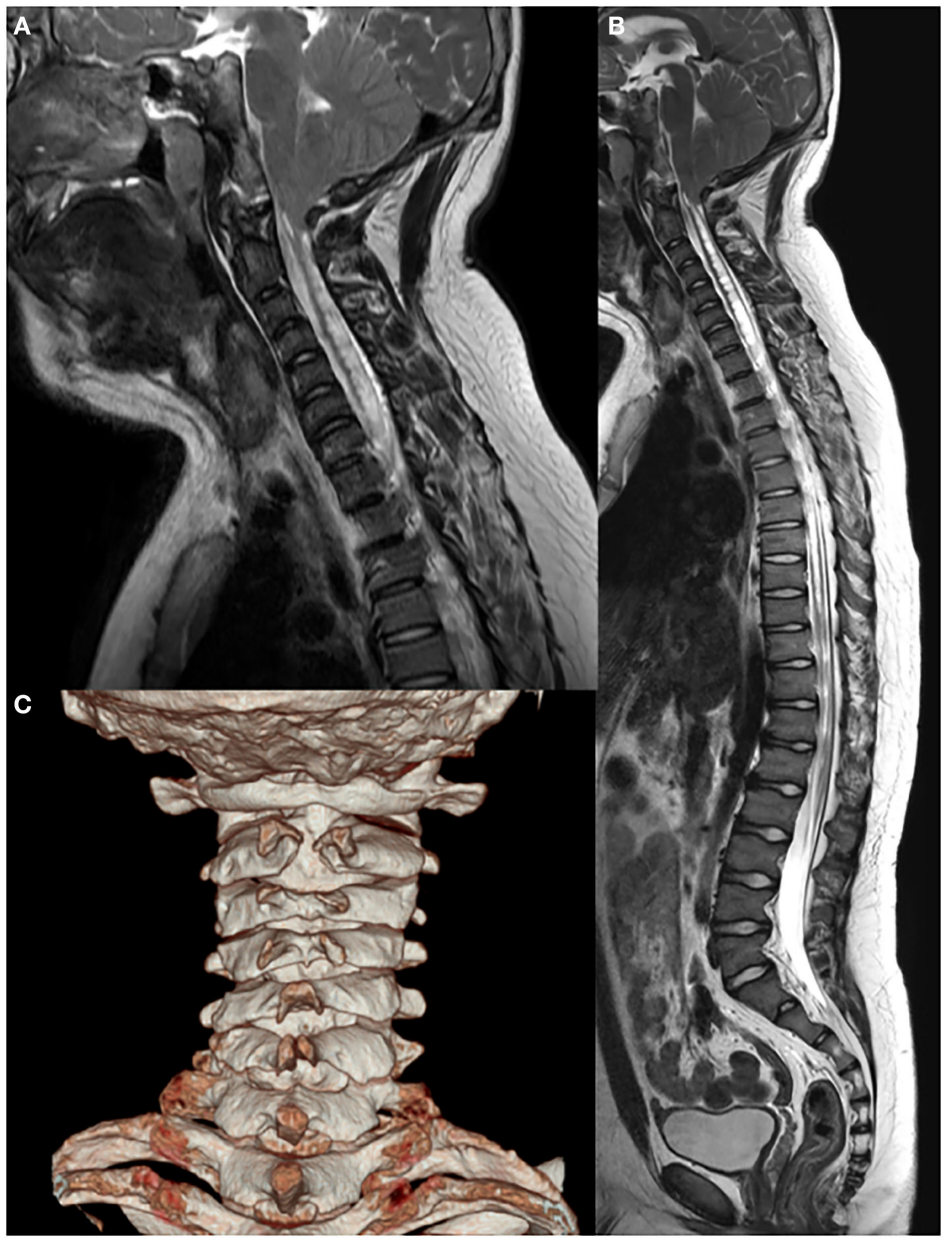
Brain and whole-spine magnetic resonance imaging and computed tomography showing **(A,B)** Chiari I malformation with extensive hydrosyringomyelia from the C1 to T12 levels and **(C)** C2 spina bifida.

Whole-spine X-ray revealed thoracic scoliosis (Cobb angle of 17°) and flattening of the vertebral body. X-ray of the upper and lower extremities revealed valgus deformity of the elbow and knee joints with mild humerus and tibia bone bowing. Computed tomography (CT) of the skull revealed irregular sclerosis with hyperostosis, obliteration of the frontal sinus, frontal bone thickening and protrusion. Three-dimensional (3D) CT of skull demonstrated premature closure of all cranial sutures (pansynostosis) (Figure 2). Brain and whole-spine magnetic resonance imaging (MRI) revealed cerebellar tonsillar inferior herniation, which clinically translates to CM1, with extensive hydrosyringomyelia from the C1 to T12 levels and C2 spina bifida occulta (Figure 3). Laboratory test, including complete blood cell count, blood chemistry, and immunochemistry, were normal. Electrodiagnostic tests, including nerve conduction study, electromyography, and somatosensory and motor-evoked potential tests, were within the normal range.

### Cytogenetic and Molecular Analyses

Chromosomal study revealed a karyotype of 46,XY without anomalies. No significant microdeletion or duplication was detected *via* the chromosomal microarray test. Targeted gene panel sequencing was performed using the Illumina MiSeqDxTM Platform (Illumina Inc., San Diego, CA, USA) with 150-bp paired-end sequencing. The targeted gene panel was custom-made and included 124 genes spanning a 461,040-bp region related to skeletal dysplasia (see Supplementary Material). Exomes were captured using a customized Target Enrichment Kit (Celemics, Seoul, Korea). The enrichment of sequenced target genes was hybridized with oligonucleotide probes. The reference genome used was hg19. Alignment was performed using BWA-mem (version 0.7.10), and variant annotation was performed using Variant Effect Predictor and dbNSFP (18, 19).

**Figure 4:**
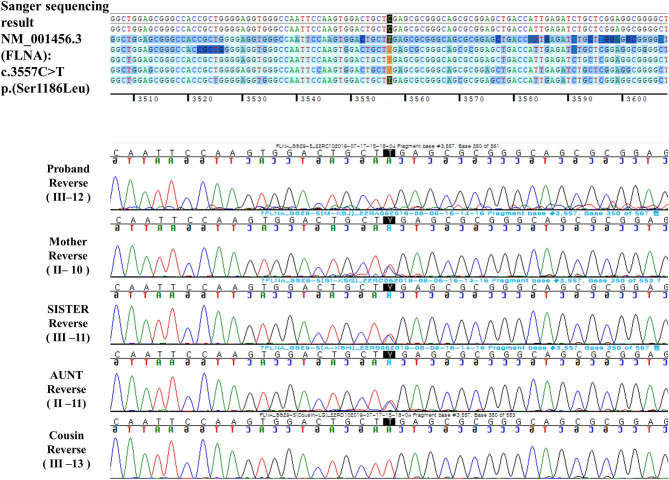
Reverse DNA sequencing chromatogram of the patients and his family members.

A hemizygous missense variant (c.3557C>T/p.Ser1186Leu) in *FLNA* (NM_001456.3), which was previously reported as being pathogenic and related to FMD1, was detected (11, 20, 21). Family gene analysis revealed that his mother, aunt, and sister (who had no symptoms) carried the same heterozygous *FLNA* variant and that his maternal male cousin with similar facial dysmorphism had the same hemizygous *FLNA* variant as the proband. Finally, the patient was diagnosed with FMD1 inherited from the asymptomatic maternal carrier (Figure 4).

## Discussion

OPDSD was first described in 1962 by Taybi, and FMD1 was reported in 1969 by Gorlin and Cohen (22, 23). Since then, various clinical features related to OPDSD have been reported. In the past, molecular genetic studies found a relationship between OPDSD and the distal Xq28 chromosome in the form of allelic variants of the *FLNA* (10, 24). The diagnosis of FMD1 is based on X-linked inheritance, clinical manifestations, and radiological studies and can be confirmed by detecting a *FLNA* pathogenic variant *via* molecular cytogenetic studies. Recently, technological advances, such as next-generation sequencing, have increased the accessibility and accuracy of diagnosis. The *FLNA* is not only associated with OPDSD caused by gain-of-function but also with loss-of-function diseases that do not cause skeletal dysplasia, such as periventricular heterotopia 1 (OMIM #300049), X-linked cardiac valvular dysplasia (OMIM #314400), and congenital short-bowel syndrome (OMIM #300048) (25).

FMD1 account for ~50% of all the FMD patients, and the other 50% are diagnosed with FMD2 or FMD3, with pathogenic variants of *MAP3K7* or *TAB2*, respectively (2). FMD1 shows X-linked recessive inheritance, whereas other OPDSDs show X-linked dominant inheritance. Dozens of unrelated families with FMD1 have been reported to date; they mostly carry single-nucleotide variants of *FLNA*, resulting in missense mutations. Among the missense mutations, the c.3557C>T transition, which causes Ser1186Leu substitution (as detected in the current patient) is the most frequent mutation. The clinical manifestations of FMD1 include hyperostosis of the skull, supraorbital hyperostosis, sensorineural and/or conductive hearing loss, hypertelorism, agenesis of the sinuses in the skull, oligohypodontia, distal phalangeal hypoplasia, progressive joint contracture, scoliosis, limb bowing, shoulder girdle and hand intrinsic muscle underdevelopment, and hydronephrosis (11). FMD1 is distinguished from other OPDSDs based on the presence of a cleft palate, a joint contracture, or a tracheobronchial tree malformation (3). To date, only one case of CNS involvement, i.e., a CM1, has been reported in FMD1 in 1999 (26). However, the author was unsure whether the CM1 was a characteristic of FMD1 or an incidental finding because it was the first report of such an occurrence. In addition, many previous reports of FMD1 did not mention whether brain MRI was performed, and it is not clear whether CM1 was present. Because CM1 was observed in our patient with FMD1, there is a possibility that this type of malformation is a manifestation of this disease.

CM1 is defined as a cerebellar tonsillar herniation below the foramen magnum >5 mm. The common neurological symptoms caused by CM1 include headache, gait disturbance, weakness, sensory disturbance, poor coordination, or hypo- or hyper-reflexia. Two-third of the patients with CM1 show small and shallow posterior fossa and foramen magnum overcrowding, possibly resulting from craniocervical bony dysplasia caused by a paraxial mesodermal defect (27–29). Moreover, CM1 can be observed as secondary to other pathology causing increased intracranial pressure, overcrowding in the posterior fossa with space-occupying lesions, or lower intrathecal pressure (e.g., leaking of spinal fluid or lumbar-to-peritoneal shunt) (30). Bidot et al. revealed that idiopathic intracranial hypertension can be accompanied by CM1 (31). In addition, CMs have been reported in patients with craniosynostosis similar to that in our patient. Cinalli et al. reported that ~70% of the Crouzon's syndrome, which is characterized by craniosynostosis, had CMs, particularly in the lambdoid suture premature closure (32, 33). Several genetic studies revealed that CM1 is related to genetic factors and candidate genes include *FGFR2, PAX1, DFNB1, GDF3, CDX1, FLT1*, and *ALDH1A2* (34–37). Several syndromic disorders, such as Klippel–Feil syndrome, achondroplasia, Crouzon's syndrome, neurofibromatosis are found to be related to CM1 (38, 39). Most genetic predispositions for CM1 have been found to be caused by genetic factors related to the overcrowding of posterior fossa or underdevelopment of occipital bone. However, the genetic background is still uncertain and various factors are considered to be combined (40). Approximately 15–26% of CM1 patients suffer from lower brain stem or cranial nerve dysfunction, such as vocal cord paralysis, sensorineural hearing loss, sleep apnea, recurrent aspiration, or sinus bradycardia (41–43). Our patient also showed mild dysarthria, possibly owing to an association with cranial nerve involvement. However, sensorineural hearing loss appears to have little correlation with cranial nerve dysfunction secondary to CM1 because sensorineural or conductive hearing loss is commonly observed in patients with OPDSDs (44).

Extensive hydrosyringomyelia associated with CM1 was observed in the present case. To date, there have been no reports of hydrosyringomyelia in patients with OPDSD. Pathophysiologically, CM1 may be accompanied by syringomyelia. A study reported that 65% of patients with symptomatic CM1 exhibited syringomyelia (45). In the presence of CM1, cerebellar tonsil occlude the narrow subarachnoid space located around the foramen magnum. The propagation of existing syringomyelia may occur *via* the expansion of the brain during systole, which obstructs the subarachnoid space across the foramen magnum; moreover, the piston-like rapid movement of CSF during systole generates pressure, which propels the fluid inferiorly (46). However, the mechanism underlying syringomyelia formation remains unclear, although several hypotheses have been proposed based on the narrowed space and high subarachnoid space pressure; nevertheless, this cannot explain the rare passage between the fourth ventricle and syrinx and has not been confirmed (47).

Craniofacial anomaly is one of the major features of OPDSD, and several cases with craniosynostosis have been reported. To date, six patients with gain-of-function variants of *FLNA* have been reported to show craniosynostosis, and there was only one patient who was diagnosed with FMD1 with pansynostosis similar to our patient (48). Our case is the seventh case of craniosynostosis in OPDSD and second for pansynostosis, emphasizing that FMD1 can be associated with craniosynostosis. Therefore, if the patient is diagnosed as having gain-of-function variants of *FLNA*, craniosynostosis should be promptly checked, and if necessary, immediate surgical treatment can prevent CM1 and thus, complications such as syringomyelia.

Several reported FMD1 cases exhibit hypoplasia of hand intrinsic muscles (11). Similarly, the strength of the hand intrinsic muscles was graded as 2/5 in our patient. The development of hand weakness has not been explored in previous studies. Therefore, it is currently difficult to determine whether extensive syringomyelia, as observed in our patient, is the direct cause of hand weakness or whether hand weakness can appear without any CNS lesions because it is a clinical characteristic of FMD1. Additional research is warranted to address this issue.

OPDSD is an allelic condition, and the mutations identified to date are in-frame mutations that preserve the reading frame and length of the translated filamin A protein. Robertson et al. performed genotypic and phenotypic correlation in 41 unrelated patients with OPDSD (10). All five disorders of OPDSDs were caused by 17 mutations in four regions of *FLNA*. All mutations resulted in OPD1 and OPD2 were located in the calponin homology domains of the N-terminal actin-binding domain of *FLNA*. Some common and frequent mutations were 620C>T in OPD1, 760G>A in OPD2, and 3562G>A and 3596C>T in MNS. In addition, patients with the same mutations showed similar phenotypes. For example, four males with OPD2, possessing the 760G>A mutation, exhibited omphalocele and perinatal death. However, although they carried the same mutation, two of four patients showed hydrocephalus, while the others did not (10). Our patient exhibited CM1 with syringomyelia; however, CNS anomalies were not reported in other patients with FMD1 who carried the same missense mutation. The genotypic and phenotypic correlation in patients with OPDSD is complex and difficult to define. Further genotypic and phenotypic correlation studies are needed to address these issues.

## Conclusion

We reported a rare case of FMD1 resulting from a pathogenic variant (c.3557C>T) of *FLNA*. This case was distinguished from those previously reported in that the patient had pansynostosis and showed CNS involvement in the form of Chiari I malformation accompanied by extensive syringomyelia. If OPDSD is diagnosed, evaluation of craniosynostosis and CM1 malformation may be essential and proper treatment is critical for the prognosis of these patients. Further studies are warranted to determine whether CNS involvement is a phenotype of FMD1.

## Data Availability Statement

The original contributions presented in the study are included in the article/Supplementary Material, further inquiries can be directed to the corresponding author.

## Ethics Statement

The studies involving human participants were reviewed and approved by the Catholic University of Korea, Incheon St. Mary's hospital, South Korea. Written informed consent to participate in this study was provided by the participants' legal guardian/next of kin. Written informed consent was obtained from the parent of the patient for publication of this case report.

## Author Contributions

JK: acquisition of data, analysis and interpretation of data, writing, and critical revision of manuscript. D-WL: acquisition of data, analysis, and interpretation of data. D-HJ: study concept and design, acquisition of data, analysis and interpretation of data, study supervision, and critical revision of manuscript for intellectual content. All authors contributed to the article and approved the submitted version.

## Conflict of Interest

The authors declare that the research was conducted in the absence of any commercial or financial relationships that could be construed as a potential conflict of interest.
