# Supplementary material for: Case Report: Pansynostosis, Chiari I Malformation and Syringomyelia in a Child With Frontometaphyseal Dysplasia 1
Source: Front Pediatr. 2021 Jul 1;9:574402. doi: 10.3389/fped.2021.574402 (PMC8280522; doi:10.3389/fped.2021.574402)
Supplement: Supplementary file 1 [file Data_Sheet_1.DOCX]

Supplementary Material

## List of Skeletal dysplasia gene panel

ALPL, ARSE, COL10A1, COL1A1, COL1A2, COL2A1, COL9A1, COL9A2, COL9A3, COMP, CRTAP, CTSK, EBP, EXT1, EXT2, FGF23, FGFR1, FGFR2, FGFR3, FLNB, GNPAT, MATN3, P3H1, PEX7, PHEX, RUNX2, SLC26A2, SLC34A3, TGFB1, TRPS1, ADAMTS10, AGPS, ATP6V0A2, B3GALT6, B4GALT7, BGN, BRAF, CBL, CDC6, CDT1, CHST14, COL11A1, COL3A1, COL5A1, COL5A2, CREBBP, CUL7, DHCR7, DLL3, DYNC2H1, EP300, EVC, EVC2, FBN1, FBN2, FGD1, FKBP10, FLNA, GH1, GHR, GHRHR, GLI2, GLI3, GNAS, HESX1, HRAS, HSPG2, IFITM5, IFT80, IGF1, IGF1R, INPPL1, KRAS, LBR, LHX3, LIFR, LTBP2, LZTR1, MAP2K1, MAP2K2, NEK1, NF1, NIPBL, NRAS, NSDHL, OBSL1, ORC1, ORC4, ORC6, PCNT, POR, POU1F1, PPIB, PROP1, PTPN11, PYCR1, RAF1, RIT1, RMRP, ROR2, SBDS, SERPINH1, SHOC2, SKI, SLC35D1, SLC39A13, SMAD3, SMARCAL1, SMC1A, SMC3, SOS1, SOX3, SOX9, SRCAP, TGFB2, TGFB3, TGFBR1, TGFBR2, TRIM37, TRIP11, TRPV4, TTC21B, WDR19, WDR35

🡪 Total 124 genes (461,040-bp)
